# Platelet-derived lysophosphatidic acid mediated LPAR1 activation as a therapeutic target for osteosarcoma metastasis

**DOI:** 10.1038/s41388-021-01956-6

**Published:** 2021-07-23

**Authors:** Satoshi Takagi, Yuki Sasaki, Sumie Koike, Ai Takemoto, Yosuke Seto, Mizuki Haraguchi, Takao Ukaji, Tokuichi Kawaguchi, Minoru Sugawara, Masanori Saito, Yuki Funauchi, Keisuke Ae, Seiichi Matsumoto, Naoya Fujita, Ryohei Katayama

**Affiliations:** 1 Division of Experimental Chemotherapy, Cancer Chemotherapy Center, Japanese Foundation for Cancer Research, Tokyo, Japan; 2 Project for Development of Genomics-based Cancer Medicine, Cancer Precision Medicine Center, Japanese Foundation for Cancer Research, Tokyo, Japan; 3 Department of Orthopedic Oncology, Cancer Institute Hospital, Japanese Foundation for Cancer Research, Tokyo, Japan; 4 Sarcoma Center, Cancer Institute Hospital, Japanese Foundation for Cancer Research, Tokyo, Japan; 5 Cancer Chemotherapy Center, Japanese Foundation for Cancer Research, Tokyo, Japan

**Keywords:** Cancer microenvironment, Metastasis, Sarcoma

## Abstract

Osteosarcoma is the most common primary malignant bone cancer, with high rates of pulmonary metastasis. Osteosarcoma patients with pulmonary metastasis have worse prognosis than those with localized disease, leading to dramatically reduced survival rates. Therefore, understanding the biological characteristics of metastatic osteosarcoma and the molecular mechanisms of invasion and metastasis of osteosarcoma cells will lead to the development of innovative therapeutic intervention for advanced osteosarcoma. Here, we identified that osteosarcoma cells commonly exhibit high platelet activation-inducing characteristics, and molecules released from activated platelets promote the invasiveness of osteosarcoma cells. Given that heat-denatured platelet releasate maintained the ability to promote osteosarcoma invasion, we focused on heat-tolerant molecules, such as lipid mediators in the platelet releasate. Osteosarcoma-induced platelet activation leads to abundant lysophosphatidic acid (LPA) release. Exposure to LPA or platelet releasate induced morphological changes and increased invasiveness of osteosarcoma cells. By analyzing publicly available transcriptome datasets and our in-house osteosarcoma patient-derived xenograft tumors, we found that LPA receptor 1 (LPAR1) is notably upregulated in osteosarcoma. *LPAR1* gene KO in osteosarcoma cells abolished the platelet-mediated osteosarcoma invasion in vitro and the formation of early pulmonary metastatic foci in experimental pulmonary metastasis models. Of note, the pharmacological inhibition of LPAR1 by the orally available LPAR1 antagonist, ONO-7300243, prevented pulmonary metastasis of osteosarcoma in the mouse models. These results indicate that the LPA–LPAR1 axis is essential for the osteosarcoma invasion and metastasis, and targeting LPAR1 would be a promising therapeutic intervention for advanced osteosarcoma.

## Introduction

Osteosarcoma is a tumor of mesenchymal origin that constitutes the most common primary malignant bone cancer, exhibiting heterogeneous histological, genetic, and molecular features [1]. The incidence of osteosarcoma is 1–3 cases per million annually, with a higher incidence in children and adolescents [2]. The pathogenesis of osteosarcoma is preceded by initial *TP53* or *RB1* gene mutations, leading to chromosomal instability, followed by secondary oncogenic mutations and the development of a polyclonal disease with metastasis [1, 3]. Given that osteosarcoma is a rare malignant tumor that is highly heterogeneous, the patient survival rate has not improved in the last 40 years, especially for metastatic osteosarcomas. The lung is the most common site for initial metastasis, with 10–20% of patients with osteosarcoma presenting pulmonary nodules at the initial diagnosis [4, 5], and more than 80% of relapses occur in the lungs [6, 7]. Although the survival rate for primary localized osteosarcoma has improved since the 1970s through the introduction of active multiagent chemotherapy with surgery [8], the survival of patients with metastasis remains poor, with a 5-year overall survival rate for osteosarcoma with lung metastasis of 19–37% [4, 7, 9–11]. The first line of chemotherapy, including high-dose methotrexate, doxorubicin, cisplatin, and ifosfamide, has been introduced [12]; however, there is no consensus on either the optimal combination or the therapeutic options for patients with metastatic or recurrent osteosarcoma [13, 14]. Therefore, understanding the mechanisms of invasion and metastasis common to osteosarcoma is essential for developing more effective therapeutic approaches for treating these patients.

Platelets are a key player in hemostasis/coagulation, inflammation, and oncogenesis [15]. The role of platelets in tumor progression is exemplified by the correlation between thrombocytosis and shorter survival for several types of cancers [16]. Numerous reports have suggested that platelets play a role in tumor malignancy, facilitating metastasis by protecting circulating tumor cells from the shear stress of the bloodstream, from immunological assault during their intravascular phase and by mediating tumor embolization in the microvasculature of secondary organs [17]. Platelets also organize these processes by releasing bioactive molecules that enhance the proliferation and motility of tumor and endothelial cells, thereby promoting tumor growth, metastasis, and angiogenesis [18–21]. Several growth factors and chemokines, such as platelet-derived growth factor, vascular endothelial growth factor, transforming growth factor-β, chemokine ligand 7 (CXCL7), CXCL4, and CXCL12 are reserved in platelet granules and are released upon platelet activation [22, 23]. These peptide mediators directly promote cell proliferation and epithelial–mesenchymal transition in several types of cancers with high receptor expression [23–25]. In addition to peptide mediators, bioactive lipid mediators, including thromboxane A2 (TxA2), sphingosine-1-phosphate (S1P) and lysophosphatidic acid (LPA), are produced and released from activated platelets to modulate inflammation, immune response, and vascular homeostasis [26–28]. Recently, S1P and LPA have also been reported to contribute to tumor malignancy [29–31]. Thus, platelet–cancer cell interactions and the subsequent release of bioactive molecules are being recognized as critical for promoting tumor progression and the acquisition of malignant characteristics in carcinomas. However, the role of platelets in pulmonary metastasis of osteosarcoma remains unknown.

In this study, we identified that osteosarcoma cells commonly exhibit high platelet activation-inducing characteristics and that the cells’ invasiveness is promoted by LPA released from activated platelets. In addition, we found that LPAR1 is notably upregulated in both osteosarcoma cell lines and our in-house osteosarcoma patient-derived xenograft tumors. LPA treatment induced a morphological change and increased invasiveness in osteosarcoma. Knockout (KO) of the *LPAR1* gene in osteosarcoma cells abolished the platelet-mediated osteosarcoma invasion and the formation of early pulmonary metastasis foci. Pulmonary metastasis of osteosarcoma was significantly suppressed due to the administration of orally available LPAR1 antagonist. To the best of our knowledge, our study therefore represents the first to report on the critical roles of LPAR1 in osteosarcoma metastasis, whose findings might provide novel therapeutic strategies for suppressing advanced osteosarcoma.

## Results

### Osteosarcoma cells commonly exhibit high platelet activation-inducing characteristics, and their invasiveness is promoted by the platelet releasate

**Fig. 1:**
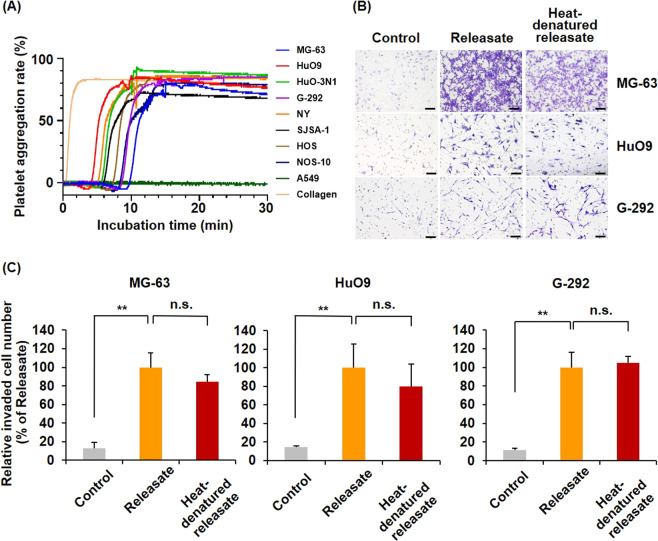
Osteosarcoma cells exhibit high platelet aggregation-inducing characteristics, and their invasiveness is promoted by the platelet releasate. **A** Platelet aggregation-inducing characteristic of osteosarcoma cells. The platelet suspension was mixed with osteosarcoma cells and incubated for 30 min at 37 °C. A549 cells and 10 μg/mL collagen were employed as negative and positive controls, respectively. The relative platelet aggregation rate was measured with a platelet aggregometer. **B**, **C** The effects of the platelet releasate on osteosarcoma cell invasion. Cells were seeded at 1 × 10^5^ cells/well in the invasion chambers and incubated for 22–24 h in the presence or absence of releasate. Heat-denatured releasate was prepared by incubating releasate at 95 °C　for 10 min using a thermal cycler. Invaded cells through the membranes were fixed and stained with crystal violet (**B** scale bars represent 100 μm). The number of invaded cells were counted and presented as percentages of the releasate values (**C**). All data are shown as means ± SD (*n* = 4). **p < 0.01 as determined by Student’s *t* test.

Platelet–cancer cell interactions and the release of bioactive molecules from activated platelets are critical for the hematogenous metastasis of carcinomas [15–19], but they are unrecognized in sarcomas. To clarify whether osteosarcoma cells interact with and activate platelets, we first measured the platelet activation ability of osteosarcoma by performing a platelet aggregation assay with eight osteosarcoma cell lines and found that all osteosarcoma cell lines exhibited higher platelet-activating characteristics than the lung adenocarcinoma cell line A549 (Fig. 1A). To investigate the effects of bioactive molecules released from activated platelets on osteosarcoma cell invasion, we harvested the reaction mixture used in the platelet aggregation assay and employed the centrifugation supernatants following the experiments as the platelet releasate (Supplementary Fig. S1). By treating the platelet releasate in lower chambers, the number of invaded MG-63, HuO9 and G-292 cells was significantly increased (Fig. 1B, C). Platelet releasate is known to be comprised of not only peptide mediators but also lipid mediators. To determine which type of mediator mainly contributed to osteosarcoma cell invasion, we heated the platelet releasate for 10 min at 95 °C, a temperature at which general proteins are heat-denatured, and we then used the resulting releasate as a chemoattractant of osteosarcoma invasion. Interestingly, the heat-denatured platelet releasate also significantly promoted osteosarcoma cell invasion (Fig. 1B, C), suggesting that heat-tolerant molecules in the platelet releasate, such as lipid mediators, would mainly contribute to the increased osteosarcoma cell invasion.

### LPAR1 is highly upregulated in osteosarcoma

**Fig. 2:**
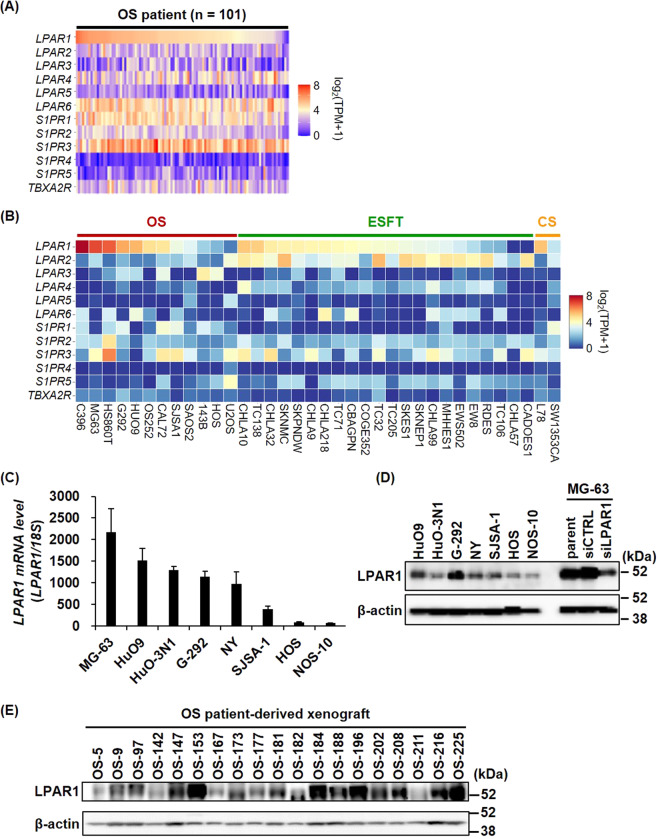
LPAR1 is highly upregulated in osteosarcoma among several types of tumors. **A** mRNA expression levels of lipid mediator receptors were analyzed using the RNA-seq data from the TARGET osteosarcoma project database (*n* = 101). **B** mRNA expression levels of lipid mediator receptors were analyzed using the RNA-seq data of bone cancer according to classification of the Cancer Cell Line Encyclopedia. OS, osteosarcoma; ESFT, the Ewing sarcoma family of tumors; CS, chondrosarcoma. **C**, **D** mRNA and protein expression levels of LPAR1 in eight human osteosarcoma cell lines. LPAR1 levels were detected by qPCR (**C**) or immunoblotting (**D**). All data are shown as means ± SD (*n* = 3). **E** Protein expression levels of LPAR1 in 19 tumor samples isolated form osteosarcoma patient-derived xenograft mice.

Lipid mediators, such as TxA2, S1P, and LPA, have reportedly been produced and released upon platelet activation, as shown by the mass spectrometry analysis of lipids in platelet releasate [32, 33]. We therefore compared the gene expression profiles of the receptors of these lipid mediators (TBA2R, S1PR1-5, and LPAR1-6) in osteosarcoma patient samples using RNA-seq data retrieved from the TARGET osteosarcoma project database. As shown in Fig. 2A, the expression levels of *LPAR1, LPAR6, S1PR1, and S1PR3* were relatively high in the osteosarcoma tumor tissues (Fig. 2A). Moreover, the expression level of *LPAR1* in osteosarcoma is much higher than that in epithelial cancers (Supplementary Fig. S2). Although there was no significant difference between non-metastatic and metastatic osteosarcomas in LPAR1 expression, metastatic osteosarcoma tended to exhibit a higher expression of LPAR1 than the other types (Supplementary Fig. S3). Since patient-derived tumor tissues represent the gene expression profiles of cancer cells and tumor-infiltrating cells, including stromal, endothelial, and immune cells, we compared the expression levels of lipid mediator receptors in the Cancer Cell Line Encyclopedia database, which reflects the gene expression profiles of cancer cells alone (Fig. 2C). Consistent with the results in Fig. 2A, *LPAR1* was upregulated in several sarcoma cell lines and showed higher expression among lipid mediator receptors in osteosarcoma. Furthermore, we confirmed that 6 of the 8 osteosarcoma cell lines, including MG-63, HuO9, HuO-3N1, G-292, NY, and SJSA-1, expressed high *LPAR1* levels by qPCR and immunoblotting analysis (Fig. 2D, E). In addition, we checked LPAR1 expression in our in-house osteosarcoma patient-derived xenograft (PDX) samples by immunoblotting with tumor lysate and found that ~80% of the PDX tumors (15/19) were LPAR1-positive (Fig. 2F).

### LPA released from activated platelets is critical for platelet releasate-mediated osteosarcoma cell migration and invasion

**Fig. 3:**
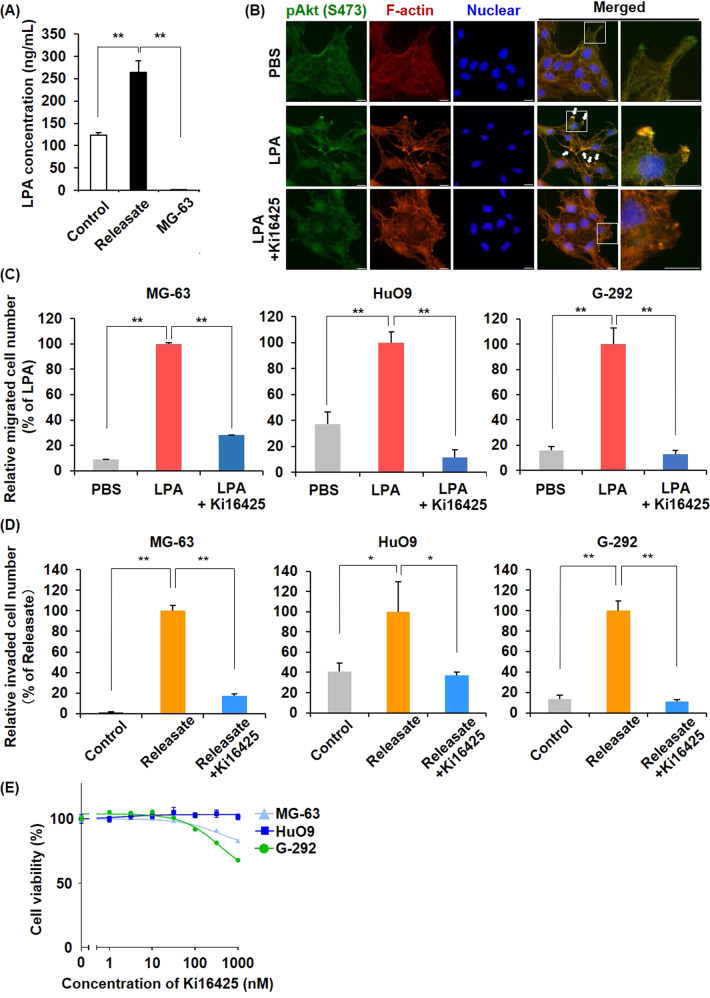
LPA released from activated platelets is critical for platelet releasate-mediated osteosarcoma cell migration and invasion. **A** LPA released from activated platelets was quantified by ELISA. Platelet suspensions (200 μL) and osteosarcoma cells (5 × 10^4^ cells/10 μL) were incubated for 30 min at 37 °C. The samples were collected in a 1.5-mL tube in the presence of 0.5 μM PGI_2_ and centrifuged at 20,000 × *g* for 5 min. The supernatants were then collected for cell treatments or ELISA. **B** Immunofluorescence staining images. MG-63 cells were starved in serum-free MEM overnight and treated with/without 10 nM LPA for 4 h. Phosphorylated Akt (green), F-actin (red) and nuclei (blue) were stained with an anti-phospho-Akt (S473) antibody, rhodamine–phalloidin reagent, and hoechst33342, respectively. Arrows indicate overlap points of phospho-Akt and F-actin. Scale bars represent 20 μm. **C** Effects of LPA treatment on osteosarcoma cell migration. Cells were seeded at 1 × 10^5^ cells/well in migration chambers and incubated for 4–6 h in the presence or absence of 10 nM LPA. In some cases, cells were pretreated with 100 nM Ki16425 for 1 h and seeded in migration chambers in the presence of 100 nM Ki16425. Migrated cells through the membranes were fixed and stained with crystal violet. The migrated cell number was counted and presented as percentages of the LPA values. **D** Effects of platelet releasate on osteosarcoma cell invasion. Cells were seeded at 1 × 10^5^ cells/well in the invasion chambers and incubated for 22–24 h in the presence or absence of platelet releasate (10 nM LPA equivalent). In some cases, cells were pretreated with 100 nM Ki16425 for 1 h and seeded in the invasion chambers in the presence of 100 nM Ki16425. Cells that had invaded through the membranes were fixed and stained with crystal violet. The invaded cell number was counted and presented as percentages of the Releasate values. **E** Effects of Ki16425 on cell proliferation. Cells were treated with a range of Ki16425 doses for 72 h. Cell viability was assessed using CellTiter-Glo Reagent. All data are shown as means ± SD (*n* = 4). **p < 0.01, *p < 0.05 as determined by Student’s *t* test.

Platelets were originally defined as major sources of LPA, producing LPA upon activation [27, 34]. Consistent with previous reports, we detected a large amount of LPA in the supernatant of the platelet-osteosarcoma reactants but not in the MG-63 cells alone by enzyme-linked immunosorbent assay (ELISA) analysis (Fig. 3A). LPARs are G protein-coupled receptors that bind LPA with varying affinities and promote signaling through specific heterotrimeric G proteins, thereby influencing the differentiation, proliferation, and motility of several types of cells [35]. In particular, LPAR1 activates three types of G proteins (G_αi/o_, G_αq/11_, and G_α12/13_), which convey signals through downstream molecules and signaling pathways, including the PI3K/AKT pathway [36]. To investigate the effect of LPA on osteosarcoma cells, we performed a time-lapse analysis and microscopic observation of osteosarcoma cells (Supplementary Video 1–4). Upon treating with LPA, we observed the formation of numerous filopodia (Supplementary Fig. S4) and co-localization of phosphorylated Akt with highly polymerized actin at the membrane protrusions (one of the characteristics of motile cells) in MG-63 and HuO9 cells (Fig. 3B, Supplementary Fig. S5). We then investigated the effects of LPA on the migration and invasion of osteosarcoma cells using chamber systems. By adding LPA to the lower chamber, we observed an increase in the number of migrated cells, which was canceled in the presence of Ki16425, a pan-LPAR antagonist (Fig. 3C, Supplementary Fig. S6). Ki16425 treatment also completely inhibited the platelet releasate-mediate osteosarcoma invasion (Fig. 3D, Supplementary Fig. S6), confirming that treatment with 100 nM Ki16425 for 72 h was not cytotoxic to the osteosarcoma cells (Fig. 3E). These results indicate that platelet–osteosarcoma cell interactions induce the release of LPA from activated platelets and that LPA included in the platelet releasate is critical for platelet releasate-mediated osteosarcoma cell migration and invasion.

### LPA–LPAR1 axis is essential for platelet releasate-mediated osteosarcoma cell invasion

**Fig. 4:**
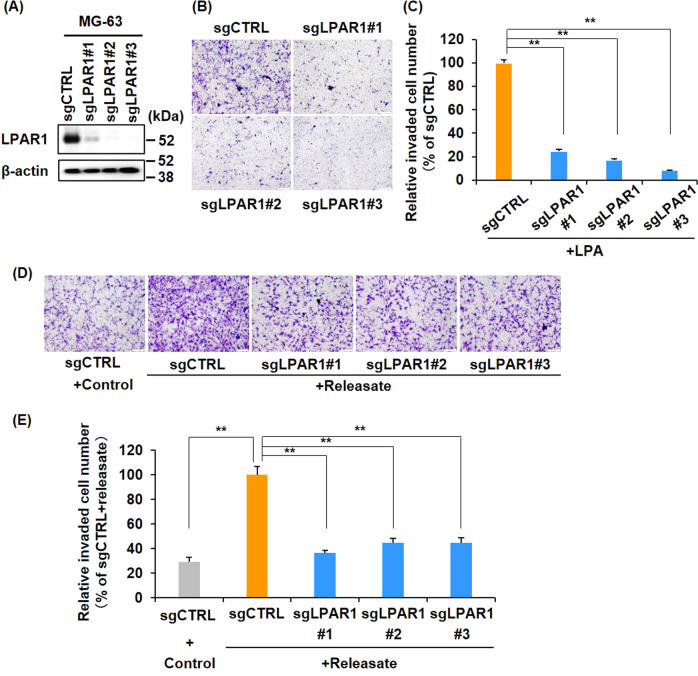
LPA–LPAR1 axis is essential for platelet releasate-mediated osteosarcoma cell invasion. **A** Establishment of LPAR1 knockout MG-63 cells. Cell lysates were immunoblotted with indicated antibodies. **B**, **C** Effects of LPA on MG-63/sgLPAR1#1-3 cell invasion. Cells were seeded at 1 × 10^5^ cells/well in invasion chambers and incubated for 22–24 h in the presence or absence of 10 nM LPA. Cells that had invaded through the membranes were fixed and stained with crystal violet (**B** scale bars represent 100 μm). The relative invaded cell number was calculated using Image J software and presented as percentages of the sgCTRL values (**C**). All data are shown as means ± SD (*n* = 4). **p < 0.01 by the Student’s *t* test. **D**, **E** Effects of platelet releasate on MG-63/LPAR1-KO cell invasion. Cells were seeded at 1 × 10^5^ cells/well in invasion chambers and incubated for 22–24 h in the presence or absence of platelet releasate (10 nM LPA equivalent). Invaded cells through the membranes were fixed and stained with crystal violet (**D** scale bars represent 100 μm). The relative invaded cell number was calculated by the Image J software and presented as percentages of the values of sgCTRL (**D**). All data are shown as means ± SD (*n* = 4). **p < 0.01 as determined by Student’s *t* test.

**Fig. 5:**
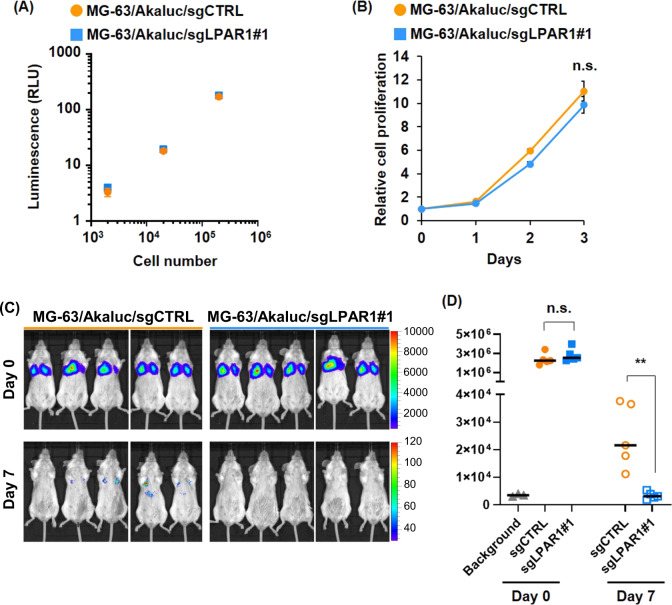
LPAR1 plays an important role in pulmonary metastasis of osteosarcoma. **A** Luminescence of Akaluc-transduced MG-63 cells. MG-63/Akaluc/sgCTRL and MG-63/Akaluc/sgLPAR1#1 cells were seeded at the indicated cell number and treated with 50 μM AkaLumine-HCl. Luminescence was measured with the Mithras LB940 Multimode Microplate Reader. **B** Cell proliferation of LPAR1 KO MG-63 cells. MG-63/Akaluc/sgCTRL and MG-63/Akaluc/sgLPAR1#1 cells were seeded and assessed by CellTiter-Glo Reagent at the indicated time points. All data are shown as means ± SD (*n* = 4). *n.s.* as determined by Student’s *t* test. **C**, **D** BLIs of mice intravenously injected Akaluc-transduced MG-63 cells. 1 × 10^6^ cells of MG-63/Akaluc/sgCTRL or MG-63/Akaluc/sgLPAR1#1 were intravenously injected into SCID-beige mice. Mice were intraperitoneally injected with 100 μL of 5 mM AkaLumine-HCL and underwent BLI. Images were taken 3 h and 7 days after cell injection (**C**), and their total flux in lung was calculated (**C**). **p < 0.01 as determined by the Mann–Whitney *U* test.

Ki16425 has been reported to inhibit LPAR1, LPAR2, and LPAR3 with a concentration of 0.34 μM, 6.5 μM, and 0.93 μM in RH7777 cells, respectively [37]. For evaluating the importance of LPAR1 in osteosarcoma cell invasion, we established polyclonal LPAR1 KO MG-63 cells, designated as sgLPAR1#1-3, using the CRISPR/Cas9 system with 3 different guide RNAs targeting LPAR1. Immunoblotting data showed that LPAR1 expression disappeared in the MG-63/sgLPAR1#1-3 cells. Moreover, the invasive cellular response to LPA was eliminated in these KO cell lines (Fig. 4B, C). Furthermore, platelet releasate-mediated MG-63 cell invasion was almost completely suppressed in the MG-63/sgLPAR1#1-3 cells (Fig. 5D, E). These results indicate that the LPA–LPAR1 axis is essential for platelet releasate-mediated osteosarcoma cell invasion.

### LPAR1 plays an important role in pulmonary metastasis of osteosarcoma

To examine the contribution of LPAR1 to the formation of early metastasis foci of osteosarcoma cells in mice models, we established Akaluc luciferase-expressing MG-63/sgCTRL and MG-63/sgLPAR1#1 cells (Fig. 5A) [38]. Because the in vitro cell proliferation rate of these cell lines was not significantly different (Fig. 5B), we next evaluated the experimental pulmonary metastasis using the in vivo imaging system after injecting AkaLumine-HCl substrate. On the day of intravenous cell injection, both cell lines were almost equally trapped in the lungs; however, only the MG-63/sgCTRL cells were engrafted in the lungs one week later and the signal intensity of MG63/sgLPAR1#1 was attenuated to the same level as that of the background (Fig. 5C, D). These results indicate that LPAR1 plays an important role in pulmonary osteosarcoma metastasis.

### Pharmacological inhibition of LPAR1 prevents pulmonary metastasis of osteosarcoma

**Fig. 6:**
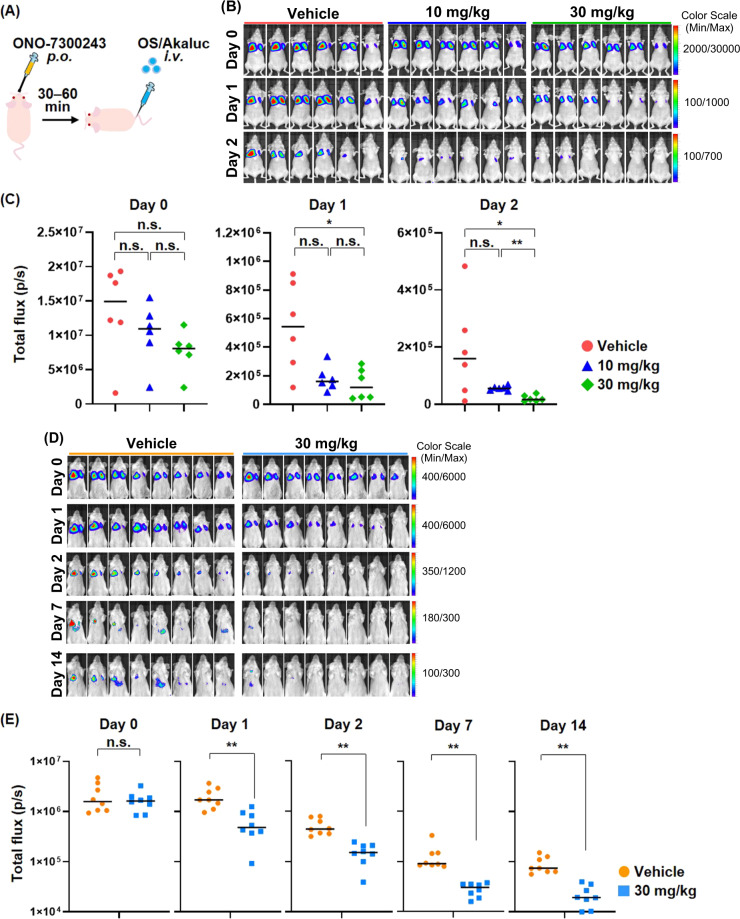
Pharmacological inhibition of LPAR1 prevents pulmonary metastasis of osteosarcoma. **A** Scheme for the animal study. ONO-7300243 was orally administrated 30–60 min before the intravenous injection of osteosarcoma cells. **B**, **C** BLIs of mice intravenously injected HuO9/Akaluc cells. 1 × 10^6^ cells of HuO9/Akaluc cells were intravenously injected into nude mice. Mice were intraperitoneally injected with 100 μL of 5 mM AkaLumine-HCL and underwent BLI. Images were taken 1.5–3 h, 1 day, and 2 days after cell injection (**B**), and their total flux was calculated by the IVIS imaging system (**C**). **D**, **E** BLIs of mice intravenously injected G-292/Akaluc cells. 1 × 10^6^ cells of G-292/Akaluc cells were intravenously injected into SCID-beige mice. Mice were intraperitoneally injected with 100 μL of 5 mM AkaLumine-HCL and underwent BLI. Images were taken 1.5–3 h, 1 day, 2 days, 7 days, and 14 days after cell injection (**D**), and their total flux in lung was calculated by the IVIS imaging system (**E**). **p < 0.01, *p < 0.05 as determined by the Mann-Whitney *U* test.

Several LPAR1 antagonists have recently been included in clinical trials for the treatment of idiopathic pulmonary fibrosis and diffuse cutaneous systemic sclerosis [39, 40]. Then, we investigated the effect of a specific pharmacological inhibitor of LPAR1, ONO-7300243, on pulmonary metastasis of osteosarcoma (Fig. 6A). Similar to the results from LPAR1 KO cells, the amount of cancer cells trapped in the lung was not significantly modified by the drug treatment on the day of cell injection; however, the pre-administration of ONO-7300243 significantly suppressed the engraftment of HuO9/Akaluc cells in the lung (Fig. 6B, C), which confirmed that treatment with ONO-7300243 for 72 h was not cytotoxic to the HuO9 cells in vitro (Supplementary Fig. S7). In the same study using another osteosarcoma cell line, G-292, the pre-administration of ONO-7300243 suppressed the tumor engraftment of osteosarcoma in the lung (Fig. 6D, E). These findings suggest that pharmacological inhibition of LPAR1 represent a novel strategy for preventing pulmonary metastasis of osteosarcoma.

## Discussion

Osteosarcoma is the most common primary malignant bone cancer with poor prognosis, which frequently leads to pulmonary metastasis. However, the molecular mechanisms of invasion and metastasis common to osteosarcoma remains poorly understood. We identified that osteosarcoma cells commonly exhibit high platelet activation-inducing characteristics. Several molecules, such as collagens, thrombin, ADP, and TBxA2, are known to induce platelet activation [17], and we previously discovered that podoplanin/Aggrus (PDPN), a type I transmembrane sialoglycoprotein expressed in several types of cancers, is a key molecule for tumor-induced platelet aggregation [41]. Because pulmonary metastasis of PDPN-positive bladder cancer cells was reduced by the knockdown of PDPN and the administration of anti-PDPN neutralizing antibodies in mice models [42], targeting platelet–cancer cell interactions would be promising for an antimetastatic approach. All osteosarcoma cell lines employed in the present study showed high platelet-activating characteristics, although several osteosarcoma cells are negative for PDPN expression (Supplementary Fig. S8), which suggests the presence of some other molecules that play central roles in osteosarcoma-mediated platelet activation along with PDPN. They might be molecular targets of anticancer metastasis agents, if the molecules are highly expressed in cancer cells and do not significantly affect the normal hemostasis.

Most cancer-related deaths are attributable to metastasis, not the primary tumor [43]. The same applies to osteosarcomas; the survival rate of patients with lung metastasis remains poor with a 5-year overall survival rate of 19–37%. In this study, we demonstrated that the pre-administration of a specific LPAR1 antagonist suppresses pulmonary metastasis of osteosarcoma cells in mice. Several LPAR1 antagonists have recently been evaluated in clinical trials for treating idiopathic pulmonary fibrosis and diffuse cutaneous systemic sclerosis [39, 40], considering that the LPA and LPAR1 roles have been demonstrated in animal models and patients [44, 45]. LPAR1 KO mice are protected from bleomycin-induced lung fibrosis by reducing the fibroblast activation and chemotaxis [44]. Similar suppressive effects were observed by the treatment of LPAR1/LPAR3 antagonists in the bleomycin models of fibrosis [46, 47]. To the best of our knowledge, our study represents the first report on the effectiveness of LPAR1 inhibition to prevent the pulmonary metastasis of osteosarcoma cells. In contrast, we did not investigate the effect of LPAR1 antagonist on host organs. Further, LPA reportedly induces endothelial cell barrier dysfunction and vascular leak [44], which could be the reason why the LPAR1 antagonist is so effective in preventing lung metastasis of osteosarcoma and slightly reducing the initial amount of cancer cells trapped in the lung.

In the present study, LPAR1 KO did not affect the in vitro cell growth and initial amount of cancer cells trapped in the lung after tail vein injection, but significantly reduced the cancer engraftment. Because platelets were originally defined as major sources of LPA based on the evidence that plasma LPA concentrations are >10-fold higher than that in serum [27, 34], suggesting that localized increases in LPA concentrations in tumor embolization occurring in the lung microvasculatures would contribute to the invasion and extravasation of osteosarcoma cells. In addition, because platelets not only circulate in the blood stream but also infiltrate into the tumor microenvironment of primary sites in several types of cancers [17], including primary bone cancers [20], LPA released from activated platelets would contribute to the detachment of osteosarcoma cells from primary sites, which is an initial early event in metastasis. Taken together, these findings provide a potential new therapeutic strategy targeting LPAR1 that could be a future therapeutic intervention for osteosarcoma.

LPAR1, LPAR2, and LPAR3 belong to the endothelial differentiation gene family which has 50–57% amino acid similarity with each other [48]. We demonstrated a notably high expression of LPAR1 and its essential role in osteosarcoma cell invasion and metastasis, whereas the expression of *LPAR3* was relatively high in some osteosarcoma cell lines (Supplementary Fig. S9). Of note, LPA treatment increased the invasiveness of HOS cells, which express very low *LPAR1* but high *LPAR3*, and it was abolished by Ki16425 treatment (data not shown). LPAR3 reportedly contribute to tumor invasion and malignancy in some types of cancers [30], and the G proteins responsible for downstream signaling of LPAR3 are G_αi/o_ and G_αq/11_, which partially overlap with those of LPAR1, G_α12/13_, G_αi/o_, and G_αq/11_ [35, 36]. In contrast, LPAR3 is a prognostic factor of ovarian cancer in the Human Protein Atlas, and its high expression is favorable in ovarian cancer. Further study is needed; however, LPAR3 might also play some roles in the invasion of osteosarcoma cells expressing low levels of LPAR1.

The development of drugs that target cancer metastasis is challenging. Matrix metalloproteinase (MMP) inhibitors were developed as antimetastatic agents on the basis of their effectiveness to suppress cancer cell invasion in preclinical models [49]. However, the phase II and phase III clinical trials of MMP inhibitors failed to exhibit therapeutic efficacy because MMP inhibitors were used for treatment in patients with advanced metastatic-stages [50]. For osteosarcoma treatment, a multi-modal approach, comprising of preoperative multidrug chemotherapy followed by local surgical therapy and then postoperative chemotherapy, has been the gold standard [51]. Although more elaborate studies are needed, LPA–LPAR1 axis might be a good target to develop therapeutic strategies to improve the prognosis of advanced osteosarcomas by preventing metastasis from the beginning of therapy using anticancer agents.

## Materials and methods

### Cell culture and reagents

Human Osteosarcoma cell lines　were purchased from American Type Culture Collection, RIKEN BioResource Research Center, or Japanese Collection of Research Bioresources Cell Bank, being used a lower passage number. MG-63, NY, and HOS cells were cultured in MEM (Wako) containing 10% FBS (CORNING), 1% nonessential amino acids solution (Wako), and 100 μg/mL Kanamycin (MEIJI). HuO9, HuO-3N1, SJSA-1, and NOS-10 cells were cultured in RPMI-1640 medium (Wako) containing 10% FBS and 100 μg/mL Kanamycin. G-292 clone A141B1 (G-292) cells were cultured in McCoy’s 5a medium containing 10% FBS. Cells were cultured at 37 °C and 5% CO_2_. The sodium salt of 1-oleoyl lysophosphatidic acid was purchased from Cayman Chemical and dissolved in ultra-pure water. Ki16425 (Santa Cruz Biotechnology) and ONO-7300243 (Cayman Chemical) were dissolved in dimethyl sulfoxide.

### Platelet isolation and aggregation assay

We collected human blood in accordance with the Declaration of Helsinki and ethics regulations, with approval by the Japanese Foundation for Cancer Research Review Board. Platelets were isolated from healthy donors who were not taking known platelet inhibitors, such as aspirin and nonsteroidal anti-inflammatory drugs, for at least 10 days prior to the blood collection. Human whole blood was collected into a sodium citrate solution (0.38%) for in vitro experiments, and obtained platelet-rich plasma (PRP) from the whole blood supernatant by centrifugation at 200 × *g* for 20 min. Prostaglandin I_2_ (PGI_2_) at a final concentration of 0.5 μM was added to the PRP, and the mixture was centrifuged at 1000 × *g* for 10 min at room temperature to separate the platelets from platelet-poor plasma (PPP) as a precipitate. Platelets were washed with modified Tyrode’s buffer (137 mM NaCl, 11.9 mM NaHCO_3_, 0.4 mM Na_2_HPO_4_, 2.7 mM KCl, 1.1 mM MgCl_2_, and 5.6 mM glucose) containing 0.5 μM PGI_2,_ and then centrifugated at 1000 × *g* for 10 min at room temperature. The washed platelet was resuspended in modified Tyrode’s buffer containing 1% PPP (2 × 10^8^ platelets/mL).

The platelet aggregation rate was measured with a platelet aggregometer (MCM Hema Tracer 313 M; SSR Engineering), as previously described [21]. Before starting the experiments, 1.2 mM CaCl_2_ was added to the platelet suspension. Cell suspension (10 μL of 5 × 10^6^ cells/mL) or PBS was added to the platelet suspension (200 μL), and then incubated at 37 °C for 30–60 min. The reactants were collected in a 1.5-mL tube in the presence of 0.5 μM PGI_2_ and centrifuged at 20,000 × *g* for 5 min. The supernatants were then collected for cell treatments or ELISA.

### In vitro invasion assay

The in vitro invasion of osteosarcoma cells were assessed according to the manufacturer’s instructions. The Corning BioCoat Matrigel Invasion Chamber (Corning) was used for the invasion assay. Cell suspension (1 × 10^5^ cells/0.5 mL) in serum-free MEM was added to the upper layer, and 0.75 mL of serum-free MEM with platelet supernatant was added to the lower layer. After incubation for 22–24 h at 37 °C, the cells on the upper surface of the membrane were completely removed by wiping with cotton swabs, and the tumor cells were fixed in 4% paraformaldehyde (FUJIFILM Wako Pure Chemical Corporation). The invaded cells on the lower surface of the membrane were stained with 1% crystal violet solution and observed under IX71 microscopy (Olympus). For the migration assay, we employed the Corning Transwell Chamber (8.0 μm pore, Corning). A cell suspension (1 × 10^5^ cells/0.3 mL) in serum-free MEM was added to the upper layer, and 1.25 mL of serum-free MEM was added to the lower layer. After incubating the solution for 4–6 h, the cells were fixed with 4% paraformaldehyde. For the chemoattractants, 10 nM LPA or platelet releasate containing 10 nM equivalent LPA (determined by ELISA) was added to the lower layer. For some experiments, we pretreated the cells with 1 μM Ki16425 1 h before harvesting and treated to the upper and lower layers.

### Detection of LPA by ELISA

The amount of LPA in the platelet releasate was measured using a human lysophosphatidic acid ELISA kit (Cusabio) according to the manufacturer’s protocol.

### Animals

Male and Female C.B-*Igh*-1b/GbmsTac-*Prkdc*^*scid*^*-Lyst*^*bg*^ N7 (SCID-beige), NOD.Cg*-Prkdc*^*scid*^*Il2rg*^*tm1Wjl*^/SzJ (NSG), and BALB/c-nu/nu mice were purchased from Charles River Laboratories Japan (Kanagawa, Japan). All animal procedures were performed using protocols approved by the Japanese Foundation for Cancer Research Animal Care and Use Committee in accordance with the relevant guidelines and regulations. In vivo bioluminescent imaging was performed with the IVIS Imaging System (PerkinElmer). Since non-specific signals were sometimes detected in the liver of AkaLumine-treated mice, the upper side of xiphoid process was subjected to the target of ROI measurement. Osteosarcoma patient-derived xenograft models were established by subcutaneously inoculating clinical samples into NSG mice. All patients provided informed consent for genetic and cell biological analyses, which were performed in accordance with protocols approved by the Institutional Review Board of the Japanese Foundation for Cancer Research (IRB approved number 2013-1092).

### Statistical analysis

The Student *t* test was performed to determine the statistical significance of the results of the in vitro experiments. The mice analysis was compared using the Mann–Whitney *U* test. Significant *P* values are shown as *P < 0.05, **P < 0.01. All statistical tests were two-sided.

## Supplementary information

Supplemental Figure

Supplemental Materials and Methods

Supplemental Video 1

Supplemental Video 2

Supplemental Video 3

Supplemental Video 4
