# Supplementary material for: Platelet-derived lysophosphatidic acid mediated LPAR1 activation as a therapeutic target for osteosarcoma metastasis
Source: Oncogene. 2021 Jul 23;40(36):5548–58. doi: 10.1038/s41388-021-01956-6 (PMC8429042; doi:10.1038/s41388-021-01956-6)
Supplement: Supplementary file 1 — Supplemental Figure [file 41388_2021_1956_MOESM1_ESM.pdf]

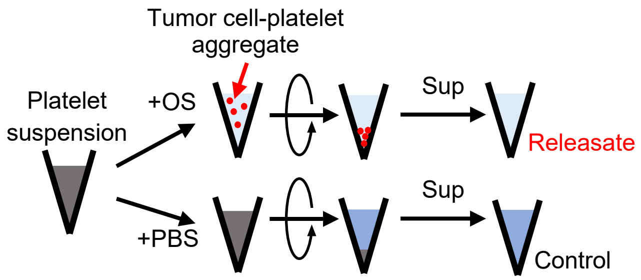

Supplemental Figure S1. Scheme for preparing the platelet releasate. Osteosarcoma cells (10 μL of 5 × 10^6 cells/mL) or PBS was added to the platelet suspension (200 μL of 2 × 10^8 platelets/mL in modified Tyrode's buffer containing 1% PPP and 1.2 mM $CaCl_2$), and then incubated at 37° C for 30–60 min by using a platelet aggregometer (MCM Hema Tracer 313M; SSR Engineering). The reactants were collected in a 1.5-mL tube in the presence of 0.5 μM PGI_2 and centrifuged at 20,000 × g for 5 min. The supernatants were then used for cell treatments or ELISA.

Supplemental Figure S2 Takagi S. *et al.*

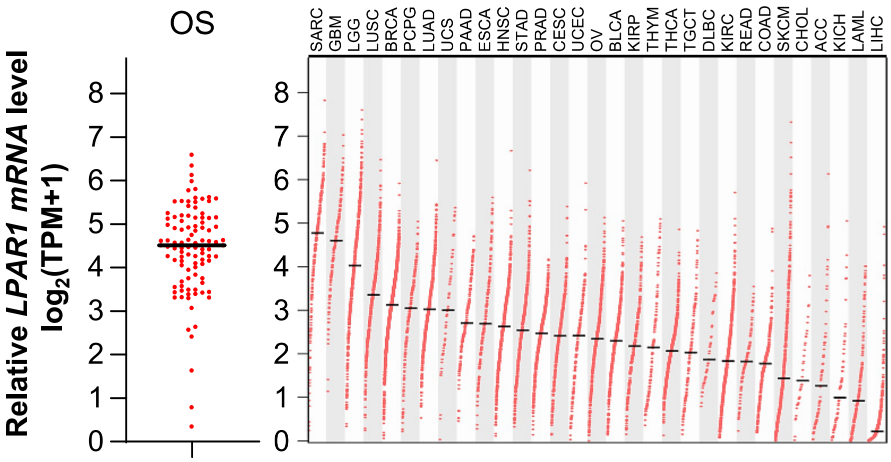

Supplemental Figure S2. LPAR1 is highly upregulated in osteosarcoma among several types of tumors. The expression levels of *LPAR1 mRNA* in patient-derived sample were analyzed using the RNA-seq data from the TARGET osteosarcoma project (left graph) and the TCGA (right graph). Bars represent mean values.

Supplemental Figure S3 Takagi S. *et al.*

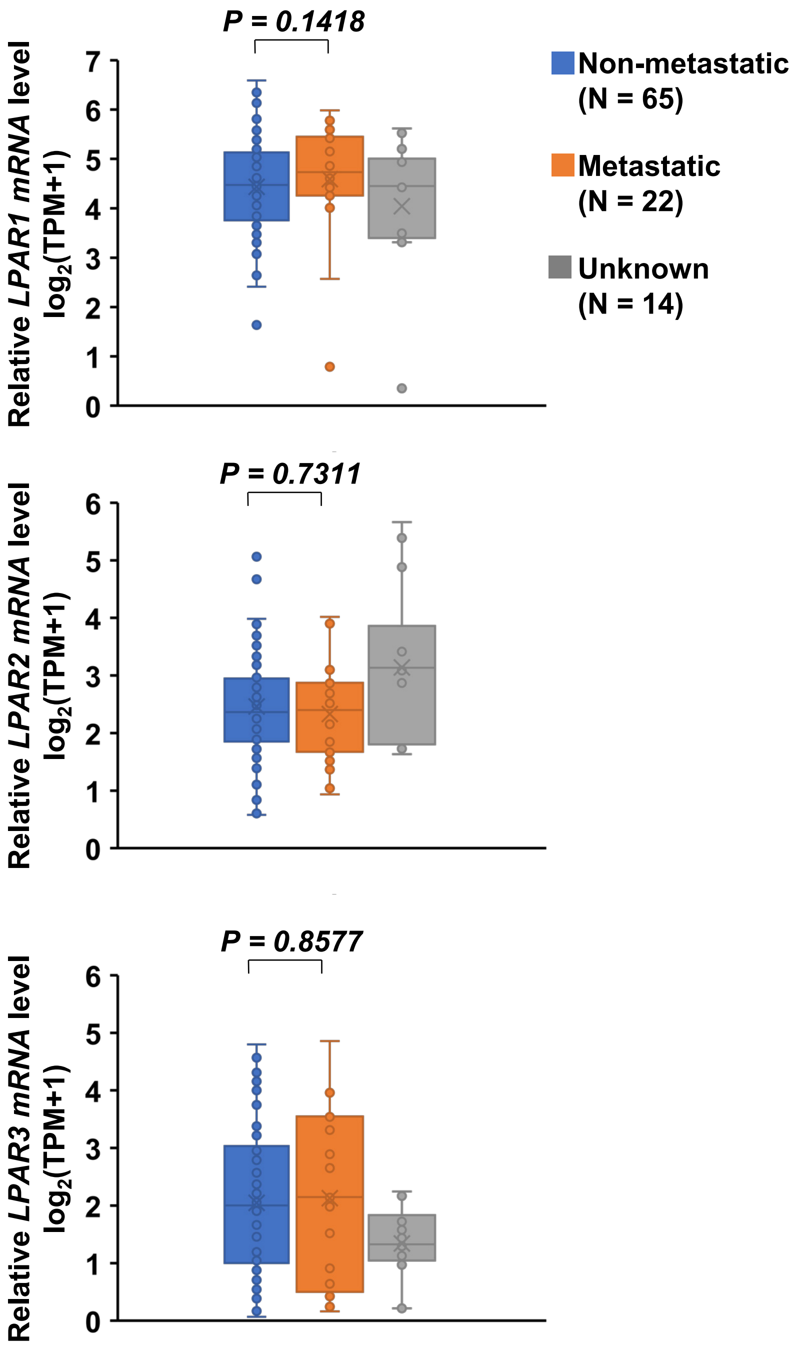

Supplemental Figure S3. The expression of LPARs in osteosarcoma and their metastatic status. The expression levels of *LPARs mRNA* in non-metastatic (N = 65), metastatic (N = 22) and unknown (N = 14) osteosarcoma. The RNA-seq data from the TARGET osteosarcoma project were used for the analysis. The data was compared using the Mann–Whitney *U* test on the $\log_2$(TPM+1) values after removing outliers.

Supplemental Figure S4 Takagi S. *et al.*

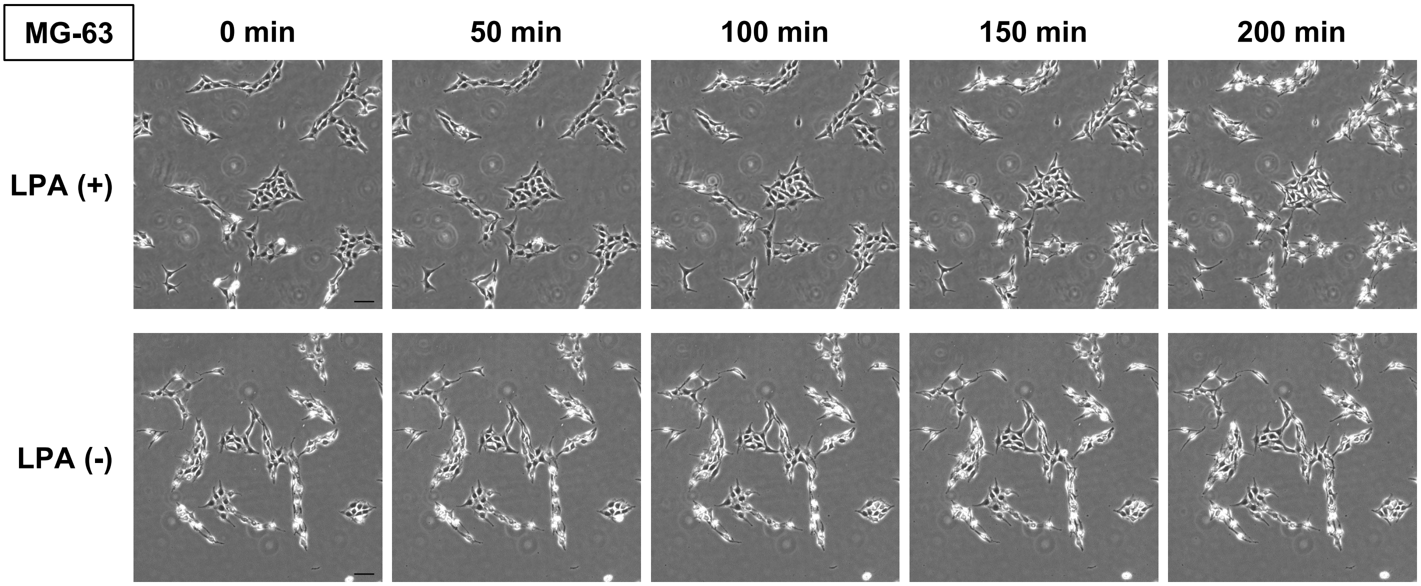

Supplemental Figure S4. Time-laps microscopic observation of MG-63 cell migration. MG-63 cells in 35-mm dish were starved overnight, and then treated with 100 nM LPA or PBS for 4 h. Morphological changes were observed by the BZ-X800 (Keyence) under 5% CO_2, at 37° C. Scale bars represent 100 μm.

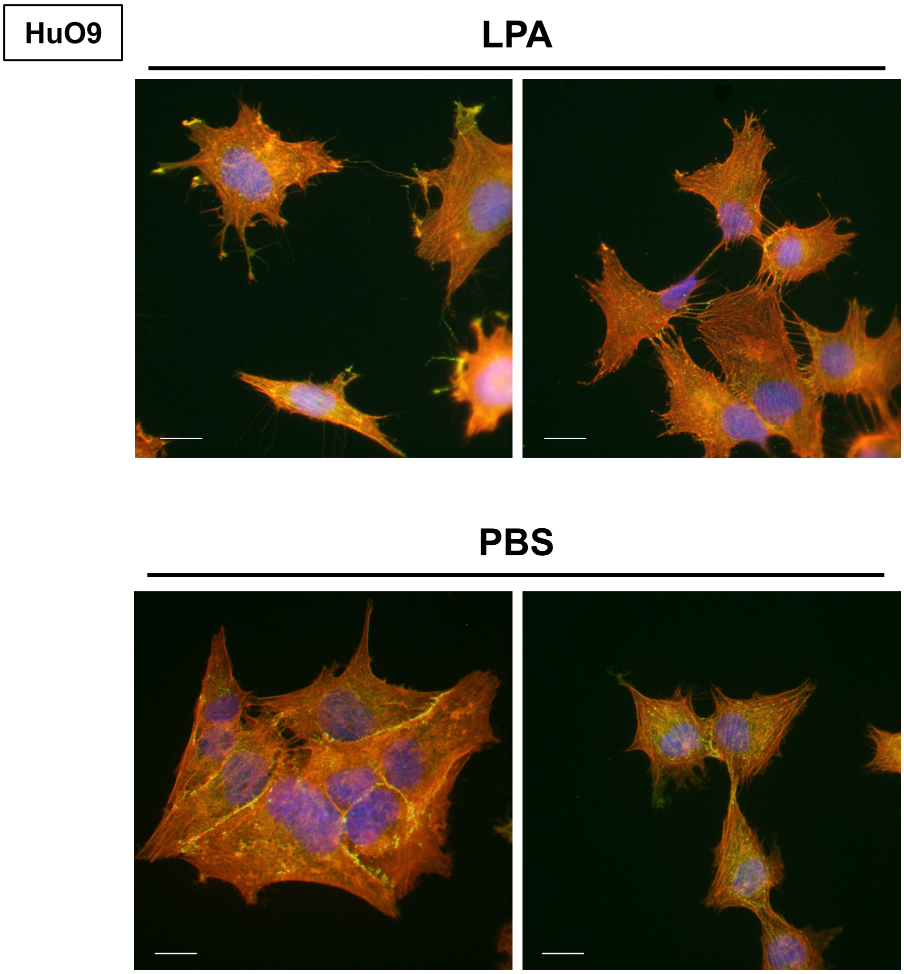

Supplemental Figure S5. Immunofluorescence images of HuO9 cells stimulated with LPA. HuO9 cells were starved in serum-free media overnight and then treated with 100 nM LPA or PBS for 4 h. After being washed with PBS, cells were fixed and stained with an anti-phospho-Akt (S473, CST#4060) antibody, rhodamine–phalloidin reagent, and hoechst33342, respectively. Two pictures taken at different points in each sample were represented. Scale bars represent 20 μm. Phosphorylated Akt (green), F-actin (red), and nuclei (blue).

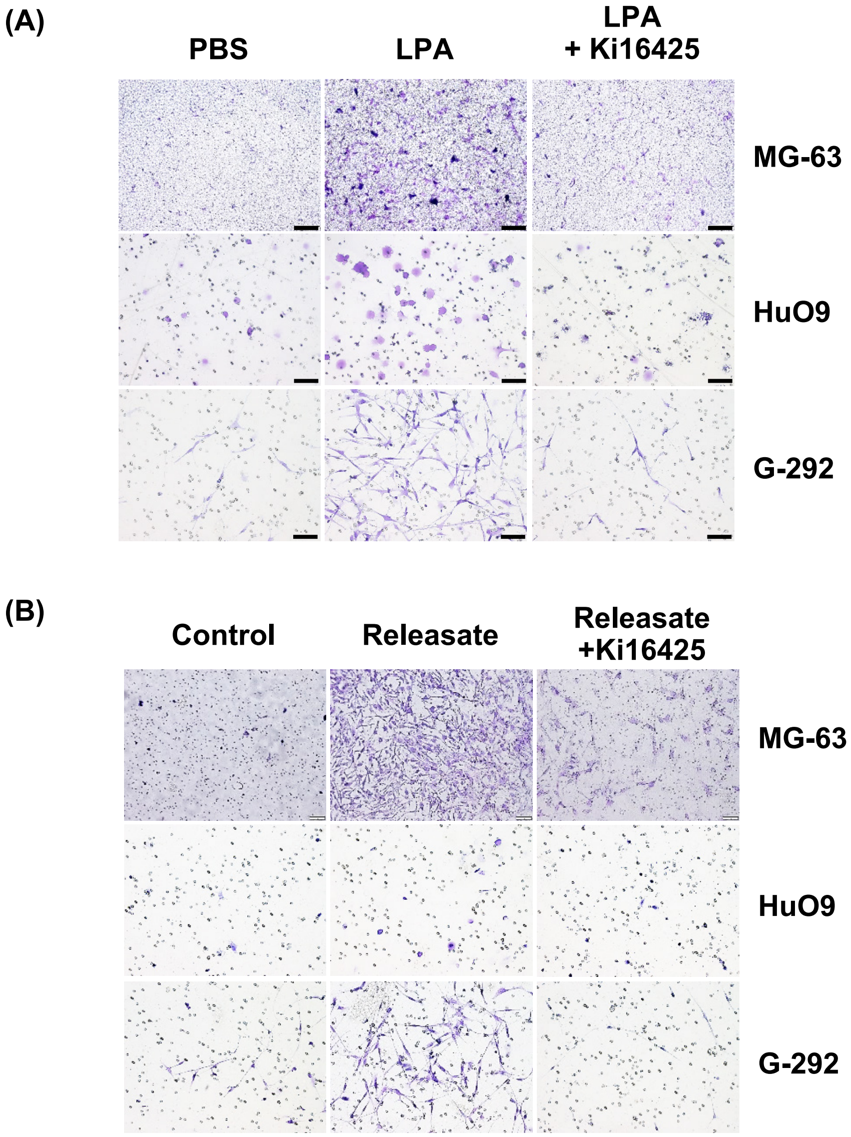

Supplemental Figure S6. Effects of LPA treatment on osteosarcoma cell migration and invasion. (A). Cells were seeded at 1 × 10^5 cells/well in migration chambers and incubated for 4–6 h in the presence or absence of 10 nM LPA. In some cases, cells were pretreated with 100 nM Ki16425 for 1 h and seeded in migration chambers in the presence of 100 nM Ki16425. Migrated cells through the membranes were fixed and stained with crystal violet. (B) Effects of platelet releasate on osteosarcoma cell invasion. Cells were seeded at 1 × 10^5 cells/well in the invasion chambers and incubated for 22–24 h in the presence or absence of platelet releasate (10 nM LPA equivalent). In some cases, cells were pretreated with 100 nM Ki16425 for 1 h and seeded in the invasion chambers in the presence of 100 nM Ki16425. Cells that had invaded through the membranes were fixed and stained with crystal violet. Scale bars represent 100 μm.

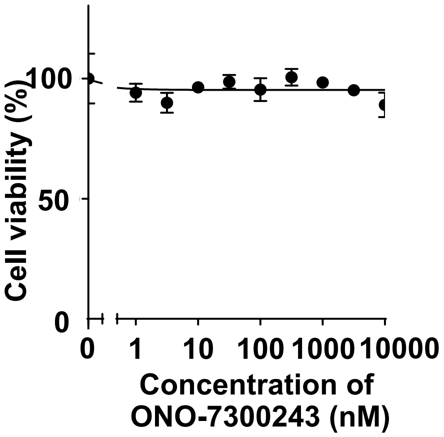

Supplemental Figure S7. Effects of ONO-7300243 on HuO9 cell proliferation. Cells were treated with a range of ONO-7300243 doses for 72 h. Cell viability was assessed using CellTiter-Glo Reagent. All data are shown as means ± SD (n = 4).

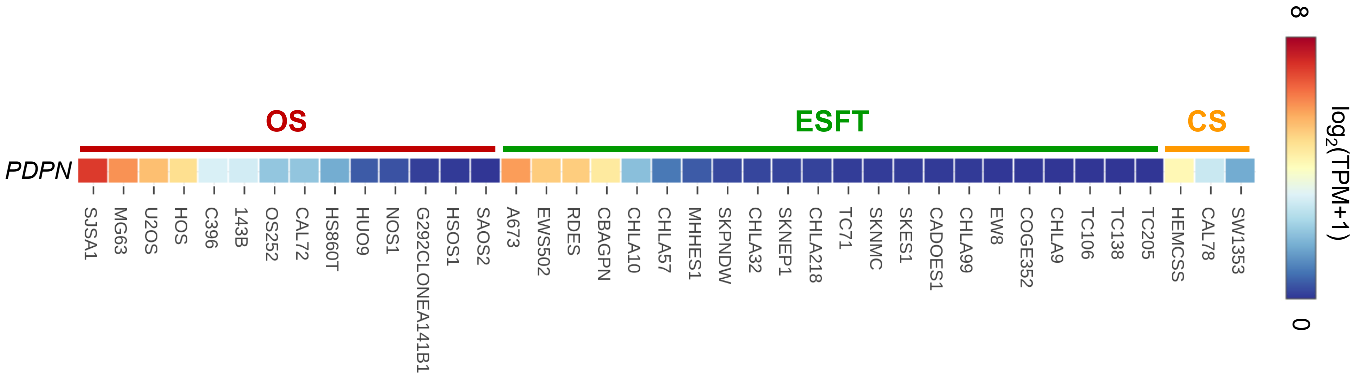

Supplemental Figure S8. *PDPN mRNA* expression in bone cancers. Heatmap showing *PDPN mRNA* levels taken from DepMap Public 20Q4. Abbreviations: OS, osteosarcoma; ESFT, Ewing sarcoma family of tumors; CS, chondrosarcoma.

Supplemental Figure S9 Takagi S. *et al.*

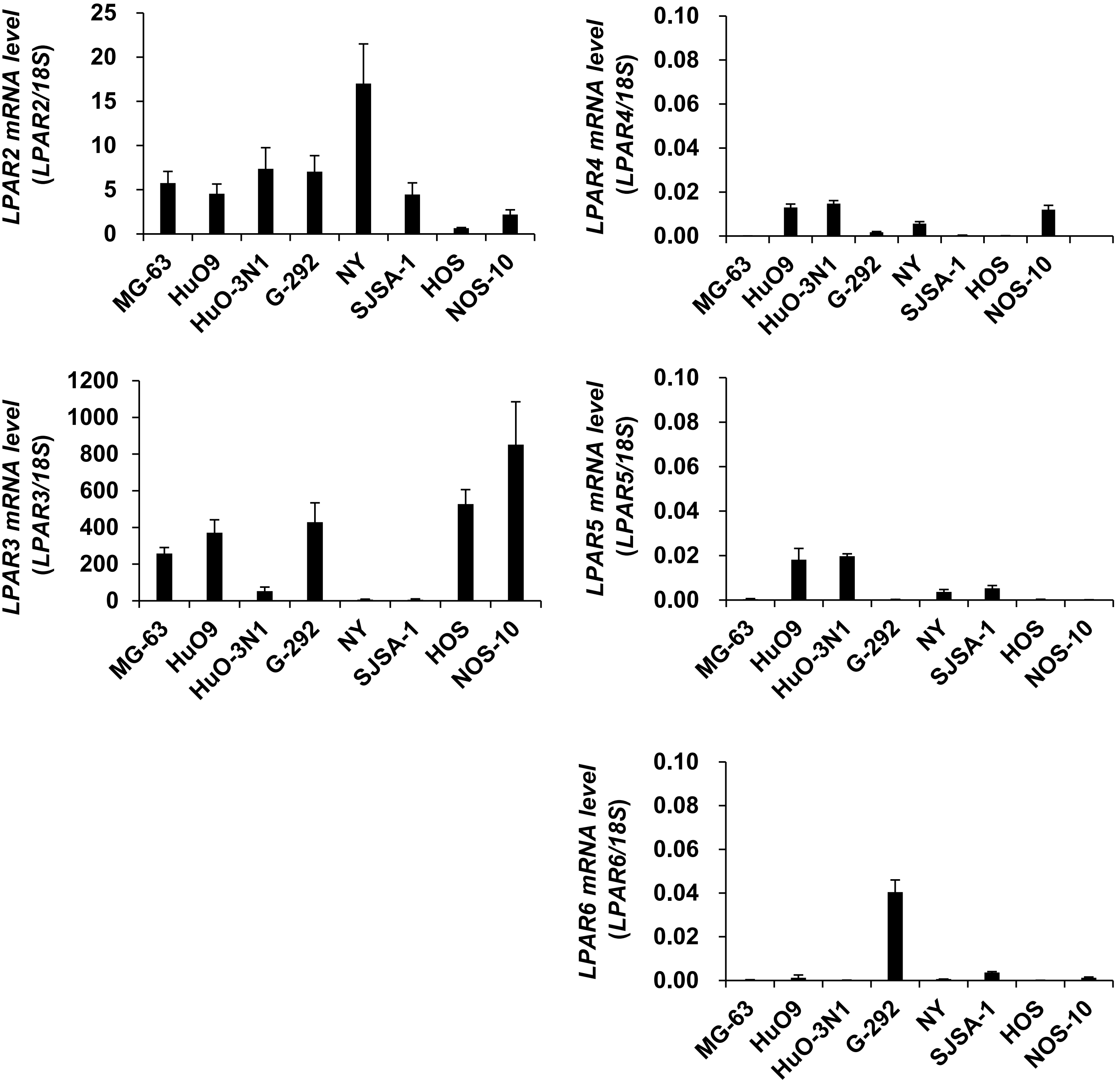

Supplemental Figure S9. mRNA expression levels of LPARs in osteosarcoma cell lines. The expression levels of *LPAR2–6* in 8 human osteosarcoma cell lines were detected by qPCR and normalized by that of 18S rRNA. The value of 5 ng of human genomic DNA was set to 1. Primer pairs used in qPCR were as follows:

human LPAR2 forward, 5′-CGCTCAGCCTGGTCAAGACT-3′;
human LPAR2 reverse, 5′-TTGCAGGACTCACAGCCTAAAC-3′;
human LPAR3 forward, 5′-AGGACACCCATGAAGCTAATGAA-3′;
human LPAR3 reverse, 5′-GCCGTCGAGGAGCAGAAC-3′;
human LPAR4 forward, 5′-CCTAGTCCTCAGTGGCGGTATT-3′;
human LPAR4 reverse, 5′-CCTTCAAAGCAGGTGGTGGTT-3′;
human LPAR5 forward, 5′-CGCAATGGCATGTGTGTTC-3′;
human LPAR5 reverse, 5′-TCCACGCTGGCTGTATATGG-3′;
human LPAR6 forward, 5′-AAACTGGTCTGTCAGGAGAAGT-3′;
human LPAR6 reverse, 5′-CAGGCAGCAGATTCATTGTCA-3′.
