# Supplementary material for: Platelet-derived lysophosphatidic acid mediated LPAR1 activation as a therapeutic target for osteosarcoma metastasis
Source: Oncogene. 2021 Jul 23;40(36):5548–58. doi: 10.1038/s41388-021-01956-6 (PMC8429042; doi:10.1038/s41388-021-01956-6)
Supplement: Supplementary file 2 — Supplemental Materials and Methods [file 41388_2021_1956_MOESM2_ESM.pdf]

Platelet-derived lysophosphatidic acid mediated LPAR1 activation as a therapeutic target for osteosarcoma metastasis

Supplemental Materials and Methods

Quantitative real-time polymerase chain reaction

Quantitative real-time polymerase chain reaction (qPCR) was performed using FastStart Essential DNA Green Master (Roche) and Light Cycler 96 (Roche) by the ΔΔCt method normalized to the 18S ribosomal RNA (rRNA) expression. Primer pairs used in qRT-PCR were as follows: human *LPAR1* forward, 5′-AATCGGGATACCATGATGAGTCTT-3′; human *LPAR1* reverse, 5′-CCAGGAGTCCAGCAGATGATAAA-3′; human *18S rRNA* forward, 5′-CTACCACATCCAAGGAAGCA-3′; human *18S rRNA* reverse, 5′-TTTTTCGTCACTACCTCCCCG-3′. Complementary DNAs were prepared with the Transcriptor First Strand cDNA Synthesis Kit (Roche) according to the manufacturer's protocols.

Immunoblot analysis

Cells were harvested using a cell scraper and lysed in lysis buffer [10 mM Tris-HCl (pH 7.4), 10 mM EDTA, 100 mM DTT, 1% Triton X-100] containing the cOmplete EDTA-free protease inhibitor cocktail (Roche) and PhosSTOP phosphatase inhibitor cocktail (Roche). The lysate was sonicated for 10–15 min on ice. The protein concentration was measured using the Pierce™ BCA Protein Assay Kit (Thermo Fisher) after centrifugation at 20,000 × g for 10 min. Equal amounts of cell lysates were electrophoresed in sodium dodecyl sulfate-polyacrylamide gel without boiling. The proteins were transferred to polyvinylidene difluoride membranes and immunoblotted with anti-human LPAR1 (#ab23698, abcam, 1:500) and β-actin (#A5441, Sigma, 1:5,000) Abs. The SuperSignal West Femto Maximum Sensitivity Substrate (Thermo Fisher) and the Amersham Imager 600 (Amersham) were used for the detection. The siRNAs targeting human LPAR1 (#LQ-003656-00-0010) and their control were purchased from the Dharmacon. Transfection of siRNAs was performed with the Lipofectamine RNAiMAX Reagent (Thermo Fisher Scientific) according to the manufacture's protocol.

Immunofluorescence

Cells were cultured on the glass bottom dishes for 3 days. After overnight serum starvation, the cells were treated with/without 10 nM LPA for 4 h, fixed with 4% paraformaldehyde phosphate buffer solution (Wako) for 15 min, permeabilized with 0.1% Triton X-100 in PBS for 10 min, and blocked with 2% BSA in PBS for 30 min. Samples were incubated with an anti-phospho-Akt mAb (Ser473, #4060, CST, 1:1,000) and rhodamine–phalloidin reagent (Cytoskeleton) for 60 min, at 37°C. The bound antibody was detected with the Alexa 488-conjugated anti-rabbit IgG (Thermo Fisher Science). The samples were then washed with PBS and incubated with Hoechst33258 (1 μg/mL) for 5 minutes. Images were acquired using the IX71 microscope (Olympus).

***In vitro* proliferation assays**

Cell viability was assessed by the CellTiter-Glo Reagent (Promega) according to the manufacture's protocol. Briefly, osteosarcoma cells were seeded in a 96-well plate at a concentration of 1 × 10^3 cells/100 μL with a range of Ki16425 doses for 72 h. Subsequently, CellTiter-Glo Reagent was treated to the cells and the luminescence was measured with the Mithras LB 940 Multimode Microplate Reader (Berthold, Germany).

Plasmid construction and CRISPR/Cas9 knockout of human LPAR1

Human *LPAR1* targeting sgRNA was designed using a web tool created by the Broad Institute of MIT and Harvard (http://crispr.mit.edu/) as follows: sgRNA targeting human LPAR1#1: TTATTACCTAATGGCTAATC, #2: TGTTTGTATCTTCATCATGT, #3: ATGGAACACAGTCAGCAAGC, and a scramble sequence. Annealed sgRNA was digested by the BsmBI restriction enzyme and cloned into the pSpCas9(BB)-2A-Puro (PX459) V2.0 plasmid (Addgene). Plasmid transfection was performed with Lipofectamine 3000 reagent (Thermo Fisher Scientific) according to the manufacturer's protocol. The plasmid pHIV-AKALuc-ZsGreen was created using the pHIV-Luc-ZsGreen (Addgene, #39196) and pcDNA3 Venus-Akaluc (provided by the RIKEN BRC through the National BioResource Project of the MEXT/AMED, Japan), and used for the lentivirus production in 293FT cells according to the manufacturer's protocol.

Supplemental Video Legend

MG-63 and HuO9 cells were seeded in 35 mm dish (Iwaki) at a concentration of 0.5 or 2 × 10^4 cells/mL and cultured for 48 h. After cell attachment, the culture medium was exchanged for serum-free medium overnight. Starved cells were treated with 100 nM LPA or PBS for 4 h. Morphological changes were observed by the BZ-X800 (Keyence) under 5% CO2, at 37°C. Images were taken at 5 min intervals. Scale bars represent 100 μm.

Supplemental Video 1: MG-63 cells treated with 100 nM LPA

Supplemental Video 2: MG-63 cells treated with PBS

Supplemental Video 3: HuO9 cells treated with 100 nM LPA

Supplemental Video 4: HuO9 cells treated with PBS
